# Catch My Drift: Building Scalable and Sustainable Models for Promoting Clinician Adherence to Evidence-Based Treatments for Eating Disorders

**DOI:** 10.2196/107039

**Published:** 2026-08-12

**Authors:** Andrea Beth Goldschmidt, Andrea Kass Graham

**Affiliations:** 1 Department of Psychiatry, University of Pittsburgh, 3811 O'Hara Street, Pittsburgh, PA, 15213, United States, 1 4126473089; 2 Department of Medical Social Sciences/Center for Behavioral Intervention Technologies, Northwestern Feinberg School of Medicine, Chicago, IL, United States

**Keywords:** fidelity, adherence, drift, supervision, artificial intelligence, implementation, sustainability, scalability, eating disorders, treatment gap

## Abstract

Lock and colleagues showed promising effects for asynchronous virtual training in family-based treatment for anorexia nervosa paired with expert case-based consultation to promote fidelity. In this commentary, we present two promising directions by which to extend these early findings: (1) using AI to scale fidelity monitoring and prevent clinician drift over time; (2) broadening the reach of virtual training models beyond private practice settings to improve the quality of eating disorders services delivered in community settings and narrow the treatment gap.

## Introduction

In their recent *Journal of Medical Internet Research* article, “Training Clinicians in Private Practice in Family-Based Treatment for Anorexia Nervosa: Randomized Controlled Trial Comparing Two Online Approaches,” Lock and colleagues [1] demonstrated that private practice mental health clinicians can be trained to deliver family-based treatment (FBT) with fidelity via virtual asynchronous training paired with expert clinical case–based consultation, with corresponding positive impacts on early weight gain in select FBT patients. The virtual training format marks a significant and long-overdue departure from traditional models of training clinicians in various psychotherapy modalities—typically multiday, in-person workshops with or without ongoing consultation and supervision, both of which present significant time and financial burdens. The authors conclude that virtual training could narrow the gap in patient access to trained and competent providers delivering evidence-based treatments for anorexia nervosa and other eating-related disorders, with corresponding downstream impacts on illness course and prognosis [2]. We agree, and we believe these promising early findings open up two additional avenues for expanding the quality and reach of eating disorders training in the community: (1) developing and evaluating scalable consultation and supervision models that will sustain treatment fidelity over time; (2) evaluating the impact of virtual training models among clinicians in non–private practice settings to increase access to and quality of care for underresourced families.

## Enhancing Scalability and Sustainability of Consultation and Supervision

A key finding in the study by Lock and colleagues [1] was that clinicians achieved high levels of FBT fidelity across 2 virtual training arms (distinguished by the level of focus on skill-building in illness externalization and agnosticism, which are central tenets of FBT), as characterized by both self and expert ratings (with the former slightly higher than the latter). Although fidelity showed modest (but significant) improvements from the end of training to the end of case consultation among the 50% who participated in the latter, suggesting that high levels of fidelity can be achieved via virtual training even *prior to* receipt of personalized case-based consultation, the sustainability of these outcomes beyond the period of active consultation was not measured. It can be challenging to evaluate long-term fidelity in the context of time-limited research protocols; however, understanding fidelity beyond periods of active case consultation is a critical issue, as clinician drift is a well-documented phenomenon when deploying evidence-based treatments for mental health conditions in the context of both efficacy [3] and effectiveness research [4] that may affect patient outcomes [5]. To fully harness the potential of virtual training models to enhance scale, enlarge the workforce, and ultimately close the treatment access gap, it is imperative to identify and evaluate consultation and supervision models that are both scalable and facilitate long-term sustainability.

AI presents a novel way to promote and measure long-term fidelity while mitigating demands on limited human resources with all the attendant costs. Indeed, work is already underway to develop and evaluate AI platforms for training clinicians in evidence-based cognitive behavioral treatment models and delivering feedback on their application to simulated patients [6-8]; as technology advances, it may be possible to use large language models to monitor and enhance fidelity in the context of recorded real-world patient interactions. Although privacy concerns and the quality of AI consultation should remain central in this future work, the early research cited above suggests that AI “consultant” feedback is feasible and acceptable to most clinicians. Further, the relative anonymity of AI feedback may be appealing to some clinicians who are reticent to receive feedback from a human consultant or supervisor. Therefore, continued investment in innovative and scalable supervision models that can be applied to different psychotherapies and clinical populations, such as those with eating disorders, is warranted.

## Enhancing Reach of Training

Lock and colleagues [1] acknowledge that an important study limitation is the exclusive focus on private practice clinicians. Although they note that there are few reasons to anticipate differential *response* to training among non–private practice clinicians, there may be important differences in the professional expectations and resources of clinicians in other health care settings that might diminish *uptake*. Indeed, in our work training community-based mental health clinicians in evidence-based treatments for eating disorders (including FBT), we have found that large caseloads, limited autonomy regarding professional decision-making, and variations in organizational support for training may be important barriers to learning new treatment models [9]. While virtual training, with or without expert consultation, may provide an especially good fit for community-based clinicians in the context of some of these barriers (eg, less flexibility to manage time in light of professional demands), it may not fully address others (eg, lack of organizational support). Furthermore, families seeking treatment through community-based health care settings often face significant logistical, financial, and cultural challenges that may make implementation of FBT more difficult for these clinicians [10]. Additional training modules may be needed to encourage FBT adoption for clinicians who serve these types of patients. For these reasons, the eating disorders field is encouraged to continue extending Lock and colleagues’ [1] promising research to mental health clinicians in non–private practice settings, with the ultimate goal of improving access to care for underresourced families and thereby mitigating patient health disparities.

Taken together, Lock and colleagues [1] are to be applauded for their efforts to expand the reach of FBT training. We hope our field will harness the rapidly evolving technology landscape to build more scalable and sustainable models to monitor and promote fidelity, and that it will continue to evaluate the utility of virtual training platforms for expanding the eating disorders workforce to meet the needs of all patients.

## Abbreviations

FBT: family-based treatment
